# Experiences of a "Back Block" Practice

**Published:** 1907-06-08

**Authors:** 


### The General Practitioners' Column.

[Contributions to this Column are invited, and if accepted will be paid for.]

### EXPERIENCES OF A "BACK BLOCK" PRACTICE.

BY ONE WHO HAS TRIED IT.

The practitioner in search of an opening has often noted with dismay the numbers of brass plates which brighten the streets of any town in the kingdom. He is apt to turn his thoughts to the wilds of Further Britain, where brass plates are replaced by painted wood or iron signs, and where he hopes to find the population under-doctored. A truthful account of one such practitioner's experiences may be of interest. If, in spite of it, others shall venture to follow my steps, I will only say, with some famous character one used to read about in one's Livy: "*Macte virtute, puer!*"

Mindful of etiquette, I called on several practitioners, of whom the only one to return my call was a recent arrival. Manners are apt to be at a discount in all new countries, where, indeed, I have known a man laughed at for taking off his hat to a lady, and have seen a woman with a baby make several ineffectual attempts to swing herself up into a coach, though a crowd of some dozen men was standing by.

In talking over my chances with established practitioners they all urged my settling anywhere else but in their own neighbourhood. This I suppose I should take as a compliment. In any case, I came to the conclusion that they were right in averring that the town was as full of doctors as any London suburb. The only new arrival who was doing really well was one who had announced his intention of adhering strictly to the line of consulting surgeon; he told me practitioners were just beginning to realise that he did not mean to steal their patients. His opinion was that the ordinary colonial practitioner was apt to be an all-round man, so that there was not much opening for specialists. He excepted the towns with universities or medical schools, but in these he said the openings for specialists were already well filled. There seemed to be a consensus of opinion that the wisest course for a newly-arrived medico was to settle in some fairly good centre in the "Back Blocks," make a reputation locally, and then remove to a large town fairly near the connection he had made. "Back Blocks" are the equivalent of the American "Backwoods," just as "New Chum" is the Australasian for the American "Tenderfoot."

The "New Chum" arrives with a heart for any fate: so I proceeded to inquire whether it is possible to obtain any sort of Government appointment in these same "Back Blocks." I found that a humane but misguided Government does undertake to subsidise doctors settling in undoctored localities. The Government takes no initiative, however: even in its own engineering camps, where men meet daily with most serious accidents in felling trees and blasting cuttings, tunnels, etc., it is many months before a surgeon is appointed. Even then I know of one instance in which the doctor was not on the colonial register, and was really only capable of rendering first aid. With regard to subsidies, the initiative is left to the doctor. His course is to call on the local magnates—stationmaster, schoolmaster, a (rare) parson, and a few leading settlers—and to suggest that a hundred miles by bridle track, ballast train, and coach to a doctor is rather hard on a broken leg and impossible for a placenta prævia. He must further enlarge upon this and that degree or diploma and this or that amount of experience, and (to descend to *terra firma* after these sublime heights) explain his willingness to settle among them if they will guarantee so much a year. If the settlers think well of him they form a committee which hunts up the inhabitants and gets each to guarantee a certain sum yearly. The committee then approaches the Government and a subsidy of some £50 or so is granted to meet the settlers' guarantee of £150, say. In many cases the settlers proceed to build the doctor a house and dispensary, and meanwhile he lives at the village "boarding house."

I found it often very difficult to get one's money, even when one had received a Settlers' Guarantee, and the Government subsidy, which never exceeds £75, is not enough to live on. It must be remembered that though meat is cheap, everything else is extremely dear. The doctor must, of course, dispense, and from a newcomer Surgical Supply Associations and druggists expect an early settlement of accounts. The difficulty of getting one's fees is due to two facts: the shifting nature of the work of most of one's young bachelor patients, and the slow development of farms. One settler on my presenting a moderate bill for a case of fracture of the skull pointed to his most juvenile calves, and promised to pay me when these should have turned into beef! Quite a number of men left the station without address before I sent in my bill at the end of the month. Many people grudged the doctor's bill who were able to afford it, simply for the reason that they came of classes which at home had been accustomed to gratuitous medical relief. The consequences of such a state of things rebound on the settlers themselves. I heard numerous complaints that this doctor was alcoholic and that injected morphia, that the third was a chemist who could only extract teeth, and that the last never made a correct diagnosis except by accident, and that then his treatment was a failure. With very hard conditions of work—long night rides along bridle tracks, with rushing rivers to swim or log-bridges to cross without a hand-rail, scorched with sun in one colony, drenched with rain in another, and frozen with cold still further south—and conditions of pay such as I have described, it stands to reason that if a man goes into such a region at all it is only because, if capable, he is young and will leave it as soon as he discovers the conditions, or because he has failed elsewhere. The remedy is for the Government to appoint and to pay doctors in every new district, recovering part at least of the cost from settlers and residents in the shape of a poll-tax.

